# Diagnostic value of systematic compression ultrasonography for the detection of unrecognized venous thromboembolism in patients admitted to an internal medicine ward for dyspnea

**DOI:** 10.1007/s11739-024-03773-7

**Published:** 2024-11-06

**Authors:** Daniele Sola, Ramona Bonometti, Giulia Comola, Giulia Francesca Manfredi, Mattia Perazzi, Filippo Patrucco, Francesco Gavelli, Massimo Scacchi, Elisa Prina, Mario Pirisi, Mattia Bellan

**Affiliations:** 1 Internal Medicine Division, Santo Spirito Hospital, Casale Monferrato, Italy; 2 Pediatric Department, Buzzi Children’s Hospital, Milan, Italy; 3 Department of Translational Medicine, Università del Piemonte Orientale, UPO, Vercelli, Italy; 4 Internal Medicine Division, “Maggiore della Carità” University Hospital, Novara, Italy; 5 Medical Department, Respiratory Diseases Unit, AOU Maggiore della Carità di Novara, Novara, Italy; 6 Laboratory of Metabolic Research, IRCCS Istituto Auxologico Italiano, Ospedale S. Giuseppe, Oggebbio, Italy; 7 UO General Medicine, Ospedale San Giuseppe, Via Cadorna 90, loc. Piancavallo, 28824 Oggebbio, VB Italy

**Keywords:** Venous thromboembolism, Deep vein thrombosis, Pulmonary embolism, Compression ultrasonography

## Abstract

The diagnosis of venous thromboembolism (VTE) is complex, and many cases of pulmonary embolism (PE) and deep vein thrombosis (DVT) go undetected despite validated diagnostic algorithms. This study evaluated the diagnostic performance of compression ultrasound (CUS) when systematically performed in patients admitted to an internal medicine department for dyspnea and/or respiratory failure. We conducted a prospective observational cohort study of consecutive adult hospitalized patients admitted for dyspnea and/or respiratory failure with at least one of the following: tachycardia (> 100 bpm), tachypnea (> 20/min), chest pain, cough, syncope, or hemoptysis. Patients with a previous diagnosis of VTE or who underwent computed tomography pulmonary angiography (CTPA) or CUS during evaluation in the emergency department were excluded. The study included 263 patients (50.2% women, average age 84 years). CUS was positive in 31 patients (11.8%); Bilateral DVT was diagnosed in two patients and unilateral DVT in 29 patients. Of these, 10 underwent CT scan, with PE confirmed in 9 cases. Using the Wells score for DVT (cut-off ≥ 2), only 8 patients (25.8%) were at high risk. The accuracy of the Wells score in identifying PE was suboptimal, as 5 of 9 patients (55.5%) with confirmed PE were in the low-risk group (three-level interpretation) and 8 (89.9%) were in the “EP unlikely” group (two-level interpretation). The systematic use of CUS as a point-of-care tool can improve the diagnostic accuracy for VTE in patients admitted to internal medicine departments with dyspnea/respiratory failure.

## Introduction

Venous thromboembolism (VTE) encompasses a spectrum of disorders involving vascular occlusion by thrombotic material, primarily fibrin. This can manifest as deep vein thrombosis (DVT) and/or pulmonary embolism (PE). Over 90% of DVT cases originate in the deep veins of the lower extremities [1–3], less common sites for DVT include the pelvic veins, portal vein, hepatic veins, cerebral venous sinuses, cardiac chambers, and deep veins of the arms or thorax, especially in cases of malignancy, thoracic outlet syndrome, or long-term presence of a venous catheter [4, 5]. DVT has an annual incidence of 100–200 cases per 100,000 inhabitants [6, 7], increasing over time due to factors like aging and prevalent thromboembolism-related conditions [8]. However, the true prevalence of venous thromboembolism (VTE) is likely underestimated; a study published in 2007 found that 34% of VTE-related deaths occurred suddenly, 59% went unrecognized or untreated (diagnosed post-mortem), and only 7% were correctly diagnosed before death [9]. Indeed, the clinical presentation of DVT is highly variable, with approximately 50% of patients being completely asymptomatic [3]. Common signs include swelling (75% of cases), pain in the thigh or calf (50%), skin discoloration, increased skin temperature, and dilation of superficial collateral veins [10].

Similarly, PE is often referred to as “the great imitator,” since it represents a diagnostic challenge due to its heterogeneous clinical presentation, ranging from silent cases to sudden death resulting from hemodynamic compromise [11]. Typically, 70% of symptomatic pulmonary embolism cases stem from lower extremity deep vein thrombosis (DVT) [1]. Silent PE accompanies about one-third of symptomatic DVT cases [12].

The diagnostic approach to VTE involves the main algorithms endorsed by the European Society of Cardiology (ESC) in 2019 [13]. These algorithms start from the clinical assessment, aiming to stratify the pretest risk of VTE using different scores, including the Wells score [14, 15], Geneva score [16–19], and Pulmonary Embolism Rule-Out Criteria (PERC) [20, 21]. Based on the result of these tools, D-dimer testing and imaging modalities such as computed tomography pulmonary angiography (CTPA) or compression ultrasonography (CUS) are used to support the diagnostic process. High-risk PE cases with hemodynamic instability require immediate bedside tests like transthoracic echocardiography (TTE), which can reveal right-ventricular dysfunction or right atrial thrombus, prompting urgent reperfusion without further testing [13].

CUS is widely used to diagnose DVT, since it is a noninvasive method, offering > 90% sensitivity and 95% specificity for proximal DVT [22]. Moreover, a positive proximal CUS in symptomatic patients with suspected PE is considered diagnostic [23]. CUS is rapid and cost-effective, with two possible approaches: two-point (proximal) and complete (full leg).

Our study addresses the persistent challenge of underestimating PE despite established diagnostic protocols. To do this, we systematically performed CUS on all the patients admitted to an internal medicine ward because of dyspnea and/or respiratory failure.

## Materials and methods

We conducted a prospective observational cohort study to assess the prevalence of unrecognized VTE in a population of 263 patients admitted for dyspnea and/or respiratory failure to an internal medicine ward at S.C.D.U. Internal Medicine, AOU Maggiore della Carità in Novara from December 2018 to December 2019 (therefore before the first cases of COVID-19 were reported in Italy). The study was conducted in strict accordance with the Declaration of Helsinki and approved by the local Institutional Review Board (IRB number: CE 189/19).

We included all the patients aged 18 or over, admitted because of dyspnea and/or respiratory failure, with at least one of the following:

- Tachycardia (heart rate > 100 bpm).
- Tachypnea (respiratory rate > 20/min).
- Chest pain.
- Cough.
- Syncope.
- Hemoptysis.

Patients were excluded from the study if they refused participation, had already received a diagnosis of venous thromboembolism (VTE), were receiving anticoagulant therapy (including heparin, warfarin, acenocoumarol, or direct oral anticoagulants), or had already undergone computed tomography pulmonary angiography (CTPA) or compression ultrasonography (CUS) during their Emergency Room work-up.

### Data collection

Thorough anamnestic and clinical assessments were performed, and demographic and clinical data were collected, including comorbidities and risk factors for VTE. Pretest scores were calculated. Blood gas analysis data, specifically arterial pressure of oxygen (paO_2_) and carbon dioxide (paCO_2_), were retrieved.

### Compressive ultrasonography

**Fig. 1:**
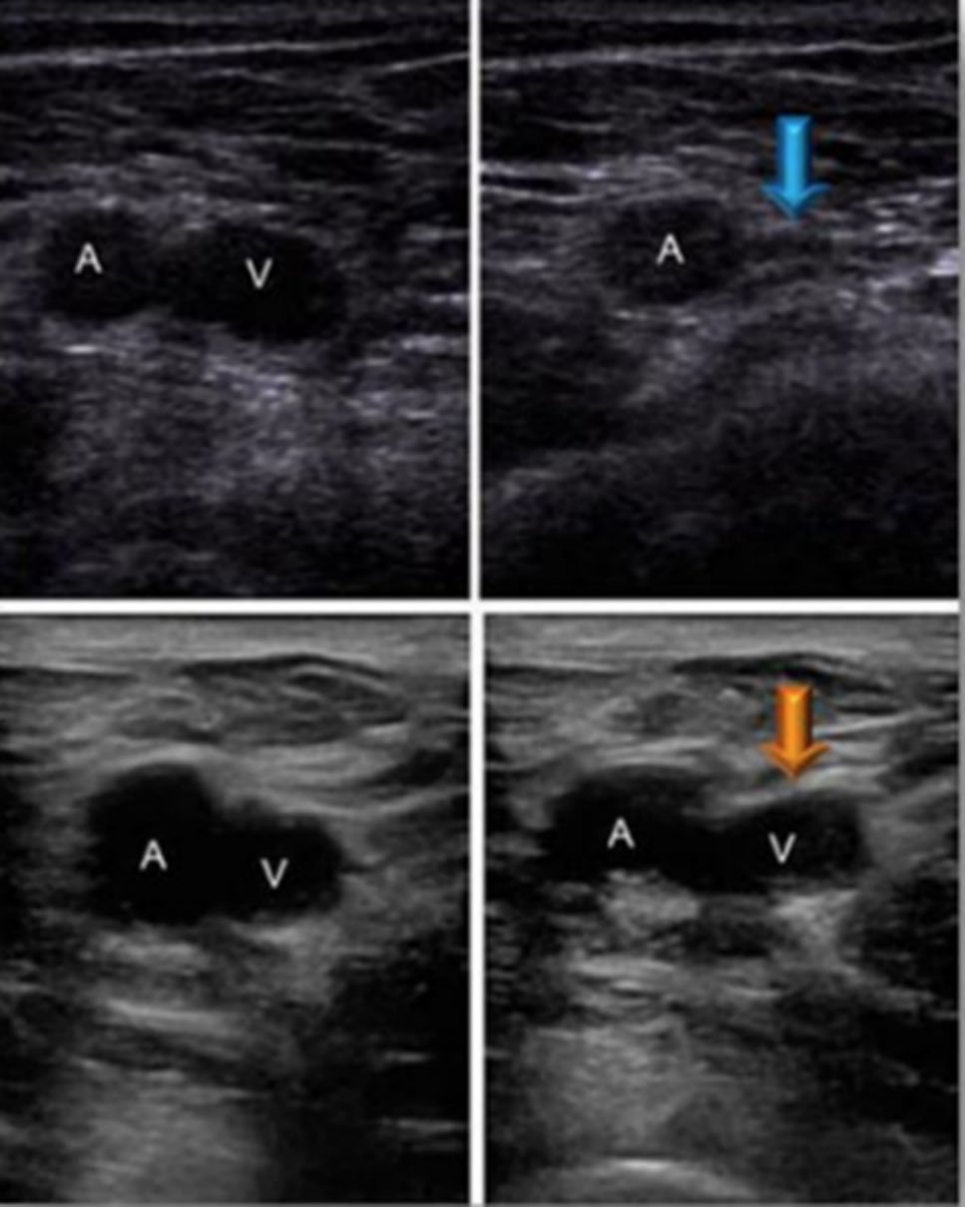
CUS interpretation. Femoral vein before and after compression. A negative CUS for DVT is identified by the blue arrow, while a positive one is represented in the panel below (orange arrow). *V* femoral vein; *A* femoral artery

For each enrolled patient, a comprehensive two-point CUS was systematically conducted within 72 h of admission to the internal medicine ward. This aimed to specifically rule out new onset VTE that might have occurred during hospital stay, acknowledging the inherent risk factor associated with hospitalization. The CUS procedures were performed with two ultrasound machines: VScan with DualProbe (GE Healthcare, USA) and Affinity 30 (Philips Healthcare, The Netherlands). With the patient lying supine, we explored the femoral and popliteal veins with a transducer ranging from 5 to 10 MHz and operating in B-Mode. Initiating the examination involved identifying the common femoral vein just distal to the inguinal ligament. The femoral vessels, situated caudally to the inguinal ligament and approximately halfway between the pubic symphysis and the anterior–superior iliac spine, were meticulously examined. The subsequent probe movement in the cranial or caudal direction aimed to locate the junction of the common femoral vein and the great saphenous vein. Direct and intermittent pressure was applied by the probe until complete compression of the examined vein. Interpretation of the results was based on the compressibility of the vein. If the vein was completely compressible, the absence of DVT in that location was inferred, resulting in a negative CUS. In contrast, an unchanged diameter indicated an incompressible vein due to occlusion, leading to a positive CUS (Fig. 1).

In cases of detected DVT through a positive CUS, further information regarding the location (femoral or popliteal) and laterality (unilateral/bilateral) was documented. Following diagnostic protocols, a positive CUS prompted additional investigations, including CTPA, to identify potential concomitant emboli in the pulmonary circulation. However, for patients with known contraindications to contrast agent, compromised renal function, pregnancy, terminal oncological conditions, or situations where clinical judgment posed a relative contraindication to CTPA, further investigation was foregone and, in such instances, anticoagulation was initiated directly based on the positive CUS.

### Statistical analysis

Collected data were stored in a computerized database and analyzed using the statistical software MedCalc (MedCalc Software Ltd, Belgio). The normality of the distribution of continuous variables was assessed using the Shapiro–Wilk test. Continuous variables are shown as medians and interquartile range.

The cut-off for statistical significance was set at *p* < 0.05.

## Results

In Table 1, we report the main features of the study population.

**Table 1:** Main demographic and clinical features of the study population

	General population N.263
Age, years	84 [75–88]
Female/male, *N* (%)	132/131 (50.2/49.8)
Signs and symptoms at hospital admission	
Dyspnea, *N* (%)	30 (96.8)
Respiratory failure, *N* (%)	27 (87.1)
Tachypnea (> 20/min), *N* (%)	19 (61,3)
Tachycardia (> 100/min), *N* (%)	27 (87.1)
Cough, *N* (%)	10 (32.3)
Chest pain, *N* (%)	2 (6.5)
Syncope, *N* (%)	2 (6.5)
Hemoptysis, *N* (%)	0 (0)
Comorbidities	
Chronic obstructive pulmonary disease, *N* (%)	94 (35.9)
Congestive heart failure, *N* (%)	80 (30.5)
Diabetes mellitus, *N* (%)	63 (24.0)
Obesity, *N* (%)	49 (19.5)
Active smoking, *N* (%)	46 (17.5)
Myeloproliferative diseases, *N* (%)	18 (6.9)
Autoimmune diseases, *N* (%)	17 (6.5)
History of stroke, *N* (%)	16 (6.1)
Inflammatory bowel disease, *N* (%)	6 (2.3)
Thrombophilia, *N* (%)	3 (1.1)
Diagnosis at hospital admission	
Pneumonia, *N* (%)	93 (35.7)
COPD exacerbation, *N* (%)	40 (15.2)
Heart failure, *N* (%)	37 (14.1)
Unspecified respiratory failure, *N* (%)	35 (13.3)
Dyspnea, *N* (%)	16 (6.1)
Other, *N* (%)	11 (4.2)
Sepsis, *N* (%)	8 (3.0)
COPD + heart failure, *N* (%)	6 (2.3)
Pulmonary edema, *N* (%)	6 (2.3)
Cancer, *N* (%)	5 (1.9)
Pleural effusion, *N* (%)	4 (1.5)

*COPD* chronic obstructive pulmonary disease

As shown, respiratory failure was prevalent at admission, affecting 86.3% of patients. The most frequent diagnoses were pneumonia (35.7%), chronic obstructive pulmonary disease (COPD) (15.2%), and congestive heart failure (CHF) (14.1%). Descriptive diagnoses such as dyspnea and unspecified respiratory failure were also notable.

A bilateral, two-points CUS was performed after a median of 24 [12–50] hours from hospital admission. CUS results were positive in 31 patients (11.8%), predominantly females (64.5%). Among the positive cases, bilateral DVT was diagnosed in two patients, while unilateral DVT was identified in 29 patients (see Table 2).

**Table 2:** Distribution of DVT in patients with positive CUS

CUS positive for DVT	Unilateral	Bilateral	Total
Femoral	25 (80.6)	0 (0.0)	25 (80.6)
Popliteal	1 (3.2)	1 (3.2)	2 (6.4)
Femoral + popliteal	3 (9.7)	1 (3.2)	4 (12.9)
Total	29 (93.6)	2 (6.4)	31 (100.0%)

The most common diagnoses among patients with a positive CUS were pneumonia (32.3%) and COPD exacerbation (12.9%); symptoms included dyspnea (96.8%), respiratory failure (87.1%), tachypnea (87.1%), and tachycardia (61.3%).

Among the 31 patients with positive CUS results, 10 underwent CT angiography, confirming pulmonary embolism in 90% of cases; therefore, a missed diagnosis of PE was definitively confirmed in 9 (3.4%) of the total patient population. The 9 patients with confirmed PE (7 females; 77.8%) had a median age of 86 [82–87] years. Initial diagnoses in the Emergency Department included pneumonia (3 patients), respiratory failure (2 patients), COPD exacerbation (1 patient), sepsis (1 patient), and other causes (2 patients).

In the context of this investigation, the diagnostic effectiveness of the Wells score for DVT, when applied to patients with positive CUS results, showed a suboptimal diagnostic performance. Indeed, using the cut-off ≥ 2, only 8 subjects (25.8%) were deemed at high risk for DVT (Table 3).

**Table 3:** The table shows, from left to right, the efficacy of the Wells score in identifying deep vein thrombosis (expressed as a number and percentage of subjects identified in relation to the total number of patients with thrombosis detected through CUS, namely 31 patients)

Wells score DVT	*N* (%)	Wells score PE	*N* (%)	Geneva score	*N* (%)	PERC criteria	*N* (%)
− 2	3 (9.7)	1	2 (22.2)	3	1 (11.1)	2	5 (55.6)
− 1	6 (19.4)	1.5	3 (33.3)	4	1 (11.1)	3	2 (22.2)
0	7 (22.6)	2.5	2 (22.2)	6	3 (33.3)	4	1 (11.1)
1	7 (22.6)	3	1 (11.1)	8	2 (22.2)	5	1 (11.1)
2	4 (12.9)	5.5	1 (11.1)	10	1 (11.1)	Total	9 (100)
3	3 (9.7)	Total	9 (100)	13	1 (11.1)		
4	1 (3.2)			Total	9 (100)		
Total	31 (100)						

Subsequent columns present the respective Wells score, Geneva score, and PERC criteria in patients receiving a diagnosis pulmonary embolism (PE) detected by computed tomography (CT scan)

Similarly, the diagnostic accuracy of the Wells score in identifying patients with PE was suboptimal, since 5 (55.5%) of patients belonged to the low-risk group according to the three-level interpretation and 8 (89.9%) belonged to the “PE unlikely” group according to the two-level interpretation.

Geneva score and PERC criteria performed better. Indeed, only one patient receiving a diagnosis of PE was in the low-risk group according to the Geneva score and all the patients showed at least one risk factor according to PERC criteria (Table 3).

## Discussion

VTE is a notable cause of hospitalization, morbidity, and mortality [11]. Despite established diagnostic protocols, PE remains under-reported [12, 24]. This study originated from the hypothesis that patients admitted to internal medicine with alternative diagnoses to VTE may harbor undetected PE, focusing on a cohort of 263 mainly elderly patients within internal medicine departments. Our results primarily suggest that the extensive and systematic use of CUS in patients admitted to an internal medicine ward because of dyspnea/respiratory failure might significantly enhance our diagnostic accuracy in this context. This finding warrants deeper insight based on the current literature.

The advanced age of this cohort provides a very realistic representation of patients in internal medicine department [25]. Our population exhibited multiple comorbidities, introducing diagnostic challenges, as these comorbidities can mimic the clinical presentation of PE, leading to respiratory failure and other signs/symptoms shared with thromboembolic conditions. Common conditions such as COPD exacerbation and heart failure were prevalent, causing dyspnea and/or respiratory failure independent of PE presence. On admission, the diagnosis of pneumonia was the most common, followed by COPD exacerbation and heart failure. Notably, patients were often admitted with generic diagnoses, highlighting the difficulty in distinguishing PE symptoms from other respiratory pathologies. All enrolled patients displayed potentially plausible clinical manifestations of PE, with 86.3% exhibiting respiratory failure on admission blood gas analysis.

The extensive use of CUS in this cohort allowed us to diagnose 31 previously undetected DVT. This is a very promising result; indeed, CUS is a cheap and safe exam, which can be repeated over time without risk for the patients. In a large multicenter study conducted by the Ultrasound Study Group of the Italian Society of Internal Medicine, which involved over 2,000 acute patients admitted to internal medicine departments, the prevalence of DVT diagnosed via CUS at admission was found to be 2.7% [26]. This percentage is notably lower than what was observed in our case series. A potential explanation for this discrepancy may be the significantly higher median age of our population (84 years) coupled with a greater prevalence of associated comorbidities, such as pneumonia and obesity. These factors likely contribute to an increased risk of DVT development in our study cohort, who were older patients and who all accessed the emergency department for dyspnea. Infectious respiratory manifestations may represent a risk factor for DVT. A meta-analysis found that among patient with COPD exacerbation, the prevalence of PE was 19.9% in outpatient and was higher at 24.7% among hospitalized patients [27]. In a retrospective analysis of 1555 patients who underwent ultrasound screening 8 weeks prior to elective surgery, an asymptomatic DVT prevalence of 10.6% in the lower limbs was observed. Consistent with our study, age over 70 years and female gender were identified as risk factors. Interestingly, the presence of malignant disease did not emerge as a risk factor [28]. Similarly, a prospective study involving 294 patients admitted to the surgical intensive care unit reported an asymptomatic deep vein thrombosis (DVT) prevalence of 7.5% upon admission. In this study, age was identified as the significant risk factor [29].

The learning curve of CUS is quick and the time needed for the examination short, making it a very useful tool at the patients’ bedside. Supporting this argument, a 2010 study involving 47 emergency room physicians is noteworthy. In this study, these physicians conducted 199 CUS on patients suspected of having thrombosis. Subsequently, these patients received a comprehensive Doppler ultrasound examination in the radiology department. The CUS displayed a sensitivity rate of 100%, successfully identifying all thromboses that were later confirmed by radiologists using the comprehensive Doppler ultrasound [22]. Furthermore, it might be feasible to transfer this competence to other health professionals, such as nurses, allowing further reduction of the burden on clinicians and empowering nurses to take care of patients. Previous papers already demonstrated the reliability of nurses performed CUS in this context, making the transfer of this skill particularly promising in the near future [30]. It might be argued that some of the DVT cases diagnosed could have been developed during hospital in-stay as a consequence of immobilization and other hospital-related risk factors. Obviously, this does not affect the relevance of making a proper diagnosis of DVT, but it would mean that VTE would not be responsible for the presenting clinical picture of the patient. However, although we could not be absolutely sure that none of the DVT developed after hospital admission, we tried to avoid this bias, performing the CUS as soon as possible, with a median interval of 24 h.

The main aim of our study was to evaluate the potential effectiveness of CUS in unveiling a missed diagnosis of PE among patients complaining dyspnea and/or respiratory failure. We must acknowledge that only a minority of those who showed a positive CUS really underwent a CTPA. This is due to the design of the study, since the decision of performing a CTPA was made upon clinical judgement, balancing the potential risk of contrast media in a population characterized by many comorbidities. Our choice was consistent with the current guidelines, which recommend accepting the diagnosis of VTE (and PE) if a CUS shows a proximal DVT in a patient with clinical suspicion of PE (class A level I of evidence) [13]. Indeed, a positive proximal CUS result has a high positive predictive value for PE, and a meta-analysis demonstrated the high diagnostic specificity (96%) along with a low sensitivity (41%) of CUS in this setting [23, 31]. Therefore, we are not aware of the real amount of missed diagnosis of PE in our cohort; however, we believe that the percentage of patients with a confirmed, missed diagnosis is not negligible (3.4%) and certainly underestimated. Indeed, there is no reason to assume that performing a systematic CTPA in all the patients with a positive CUS would have resulted in a lower percentage of positive CT scans than reported in the 10 patients who underwent the gold standard diagnostic test. Moreover, we cannot rule out that some of the patients with a negative CUS might have been affected by PE.

A possible explanation of this large amount of missed diagnosis may be the intricacies of internal medicine patients, which may impair the performance of conventional predictive scores, influencing the subjective medical judgment. Moreover, these tools are not generally applied systematically, and this may lead to an underestimation of the real risk of VTE. We therefore applied retrospectively the most often used score for the prediction of VTE to all our patients, evaluating how they would perform among those affected by VTE. Interestingly, we found that many patients with a diagnosis of DVT did not fall into the high-risk category of the Wells score for DVT. This suggests that CUS should be performed even in the context of low-risk patients.

Similarly, the Wells Score for PE did not perform very well; although we could not estimate its diagnostic performance appropriately, having excluded from the study all the patients who had already received a diagnosis of PE in the Emergency Department, we need to underscore that 5 out of 9 patients with a confirmed PE belonged to the low-risk group according to the three-level interpretation and 8 out of 9 belonged to the “PE unlikely” group according to the two-level interpretation. The Geneva score emerged as a better screening tool, albeit with potential limitations. Importantly, CUS proved superior to clinical scores in detecting unrecognized VTE.

The PERC criteria well performed in our population, but it should be kept in mind that these criteria were built to rule out the diagnosis of PE, rather to confirm it and should be therefore used in such context.

In conclusion, our study underscores the inadequacy of existing diagnostic scores in internal medicine populations, emphasizing the necessity for alternative approaches. CUS emerges as a pivotal tool, showcasing potential improvements in diagnostic accuracy for this prevalent and critical condition. Despite encouraging findings, the study acknowledges limitations, including a relatively small sample size and selective use of CT angiography, justified within the context of a "real-life" study adhering to good critical practice guidelines. Further, larger studies are required to assess the feasibility of a systematic approach to respiratory failure with compressive ultrasonography in internal medicine divisions.

## Data Availability

The raw data is available on Zenodo: 10.5281/zenodo.12192597.

## References

[1] Di Nisio M, van Es N, Büller HR (2016) Deep vein thrombosis and pulmonary embolism. Lancet (London, England) 388:3060–3073 27375038 10.1016/S0140-6736(16)30514-1

[2] Khan F, Tritschler T, Kahn SR, Rodger MA (2021) Venous thromboembolism. Lancet (London, England) 398:64–77 33984268 10.1016/S0140-6736(20)32658-1

[3] Wenger N, Sebastian T, Engelberger RP, Kucher N, Spirk D (2021) Pulmonary embolism and deep vein thrombosis: similar but different. Thromb Res 206:88–98 34454241 10.1016/j.thromres.2021.08.015

[4] Teja B et al (2024) Complication rates of central venous catheters: a systematic review and meta-analysis. JAMA Intern Med 184:474–482 38436976 10.1001/jamainternmed.2023.8232 PMC12285596

[5] Koethe Y, Bochnakova T, Kaufman CS (2022) Upper extremity deep venous thrombosis: etiologies, diagnosis, and updates in therapeutic strategies. Semin Intervent Radiol 39:475–482 36561939 10.1055/s-0042-1757937 PMC9767760

[6] Wendelboe AM, Raskob GE (2016) Global burden of thrombosis: epidemiologic aspects. Circ Res 118:1340–1347 27126645 10.1161/CIRCRESAHA.115.306841

[7] Raskob GE et al (2014) Thrombosis: a major contributor to global disease burden. Arterioscler Thromb Vasc Biol 34:2363–2371 25304324 10.1161/ATVBAHA.114.304488

[8] Amin A, Neuman WR, Lingohr-Smith M, Menges B, Lin J (2019) Venous thromboembolism prophylaxis and risk for acutely medically ill patients stratified by different ages and renal disease status. Clin Appl Thromb Off J Int Acad Clin Appl Thromb 25:1076029618823287 10.1177/1076029618823287 PMC6714996 30808218

[9] Cohen AT et al (2007) Venous thromboembolism (VTE) in Europe. The number of VTE events and associated morbidity and mortality. Thromb Haemost 98:756–764 17938798 10.1160/TH07-03-0212

[10] Alhassan S et al (2017) Clinical presentation and risk factors of venous thromboembolic disease. Crit Care Nurs Q 40:201–209 28557891 10.1097/CNQ.0000000000000159

[11] An J et al (2022) Acute pulmonary embolism and chronic thromboembolic pulmonary hypertension: clinical and serial CT pulmonary angiographic features. J Korean Med Sci 37:e76 35289137 10.3346/jkms.2022.37.e76 PMC8921210

[12] Shi Y et al (2022) Silent pulmonary embolism in deep vein thrombosis: relationship and risk factors. Clin Appl Thromb Off J Int Acad Clin Appl Thromb 28:10760296221131034 10.1177/10760296221131034 PMC9537479 36199255

[13] Konstantinides SV et al (2020) 2019 ESC Guidelines for the diagnosis and management of acute pulmonary embolism developed in collaboration with the European Respiratory Society (ERS). Eur Heart J 41:543–603 31504429 10.1093/eurheartj/ehz405

[14] Wells PS et al (1998) Use of a clinical model for safe management of patients with suspected pulmonary embolism. Ann Intern Med 129:997–1005 9867786 10.7326/0003-4819-129-12-199812150-00002

[15] Anand SS et al (1998) Does this patient have deep vein thrombosis? JAMA 279:1094–1099 9546569 10.1001/jama.279.14.1094

[16] Coelho J et al (2020) Comparison of the Wells score and the revised Geneva score as a tool to predict pulmonary embolism in outpatients over age 65. Thromb Res 196:120–126 32862033 10.1016/j.thromres.2020.07.026

[17] Shen J-H et al (2016) Comparison of the Wells score with the revised Geneva score for assessing suspected pulmonary embolism: a systematic review and meta-analysis. J Thromb Thrombolysis 41:482–492 26178041 10.1007/s11239-015-1250-2

[18] Zhao Y et al (2023) The Legend score synthesizes Wells, PERC, Geneva, D-dimer and predicts acute pulmonary embolism prior to imaging tests. Pulmonology. 10.1016/j.pulmoe.2023.10.002 37953212 10.1016/j.pulmoe.2023.10.002

[19] Le Gal G et al (2006) Prediction of pulmonary embolism in the emergency department: the revised Geneva score. Ann Intern Med 144:165–171 16461960 10.7326/0003-4819-144-3-200602070-00004

[20] Kline JA (2018) Diagnosis and exclusion of pulmonary embolism. Thromb Res 163:207–220 28683951 10.1016/j.thromres.2017.06.002

[21] Freund Y et al (2018) Effect of the pulmonary embolism rule-out criteria on subsequent thromboembolic events among low-risk emergency department patients: the PROPER randomized clinical trial. JAMA 319:559–566 29450523 10.1001/jama.2017.21904 PMC5838786

[22] Crisp JG, Lovato LM, Jang TB (2010) Compression ultrasonography of the lower extremity with portable vascular ultrasonography can accurately detect deep venous thrombosis in the emergency department. Ann Emerg Med 56:601–610 20864215 10.1016/j.annemergmed.2010.07.010

[23] Le Gal G et al (2006) A positive compression ultrasonography of the lower limb veins is highly predictive of pulmonary embolism on computed tomography in suspected patients. Thromb Haemost 95:963–966 16732375 10.1160/TH06-03-0158

[24] Valle HA et al (2020) Nonsuspected pulmonary embolism in the emergency department. Eur J Emerg Med Off J Eur Soc Emerg Med 27:379–380 10.1097/MEJ.0000000000000683 32852412

[25] Righini M, Le Gal G, Perrier A, Bounameaux H (2005) The challenge of diagnosing pulmonary embolism in elderly patients: influence of age on commonly used diagnostic tests and strategies. J Am Geriatr Soc 53:1039–1045 15935031 10.1111/j.1532-5415.2005.53309.x

[26] Loffredo L et al (2022) Asymptomatic and symptomatic deep venous thrombosis in hospitalized acutely ill medical patients: risk factors and therapeutic implications. Thromb J 20:72 36451162 10.1186/s12959-022-00433-8 PMC9709753

[27] Rizkallah J, Man SFP, Sin DD (2009) Prevalence of pulmonary embolism in acute exacerbations of COPD: a systematic review and metaanalysis. Chest 135:786–793 18812453 10.1378/chest.08-1516

[28] Hagiwara K, Watanabe Y, Suzuki T, Okamura Y, Yamashita H (2023) Prevalence of preoperative asymptomatic deep vein thrombosis in patients undergoing elective general surgery for benign disease. Ann Gastroenterol Surg 7:1042–1048 37927917 10.1002/ags3.12709 PMC10623937

[29] Harris LM et al (1997) Screening for asymptomatic deep vein thrombosis in surgical intensive care patients. J Vasc Surg 26:764–769 9372813 10.1016/s0741-5214(97)70088-0

[30] Mumoli N et al (2014) Accuracy of nurse-performed compression ultrasonography in the diagnosis of proximal symptomatic deep vein thrombosis: a prospective cohort study. J Thromb Haemost 12:430–435 24495051 10.1111/jth.12522

[31] Costa Rodrigues JD, Alzuphar S, Combescure C, Le Gal G, Perrier A (2016) Diagnostic characteristics of lower limb venous compression ultrasonography in suspected pulmonary embolism: a meta-analysis. J Thromb Haemost 14:1765–1772 27377039 10.1111/jth.13407

